# Supplementary material for: Exposure to trips and slips with increasing unpredictability while walking can improve balance recovery responses with minimum predictive gait alterations
Source: PLoS One. 2018 Sep 18;13(9):e0202913. doi: 10.1371/journal.pone.0202913 (PMC6143193; doi:10.1371/journal.pone.0202913)
Supplement: S1 Appendix — (PDF) [file pone.0202913.s001.pdf]

S1 Appendix. An analysis on free walk without gait regulation

Purpose

To examine the effects of the perturbation set up on gait speed without gait regulation.

Methods

Prior to the experiment, participant usual gait speed was measured 3 times on the electronic walkway (GAITRite ® mat, v4.0, 2010 CIR Systems, USA). An average of the usual gait speed was used to regulate participant gait during training. However, to examine the effects of the perturbation set up on gait speed, two free walks (F1 and F2 in Table 1) on the perturbation walkway without gait regulation devices were included during which participants were instructed that they may encounter a perturbation. Gait speed during the free walks trials (F1 and F2) were compared to N1 with gait regulation. Potential changes in gait speed between the two free walk trials without regulation were also examined (F1 vs F2). Paired *t*-tests were used for the above comparisons.

An extract from Table 1 The study protocol.

Condition 1: Right leg / fixed location
**F1**, **N1**, S1, T1, **F2**, N2, S2, T2, S3, T3, S4, T4

F: free walk (no regulation), N: non-perturbed walk (regulated to usual gait speed), S: slip, T: trip. The bolded letters signify the trials used to in the analyses.

Results

During the first free walk (F1: 1.30 ± 0.21 m/s), participants walked significantly slower compared with the regulated non-perturbed walk (N1: 1.44 ± 0.20 m/s) (p<0.001, Fig 6). The second free walk that followed the first slip and trip trials (F2: 1.37 ± 0.23 m/s) participants walked faster than during the first free walk (F1, p=0.043), yet significantly slower than regulated walk (N1) (p<0.001).

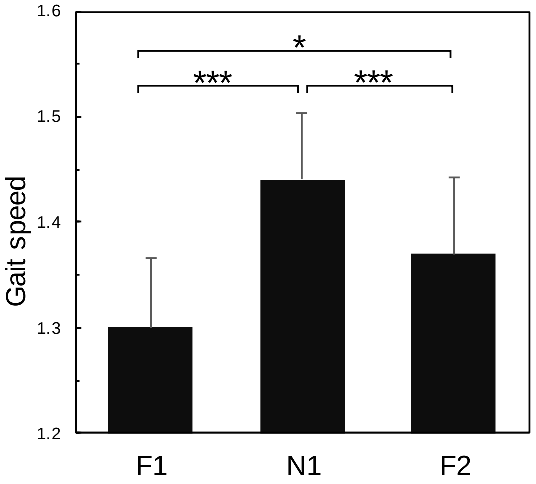

Fig 6. Gait speed during free walks (F1 and F2) and regulated walk (N1).
* $p < 0.05$, *** $p < 0.001$

Discussion

The significantly slow gait speed at F1 on the walkway may be due to anxiety or caution associated with the possible hazards even before the first exposure to perturbations. The result that the free gait speed significantly increased from F1 to F2 was contrary to our expectation; that it would slow down after the first exposure to a slip and a trip. However, the increased gait speed from F1 to F2 may be explained by a residue effect of the gait regulation from previous trials (N1, S1 and T1). In any case, F1 and F2 gait speed were both significantly slower than N1 (participants usual gait speed measured using an electronic walkway). These observations confirmed that gait regulation was required to maintain participant’s gait pattern consistent and examine the changes in reactive balance control.
