# Supplementary material for: Exposure to trips and slips with increasing unpredictability while walking can improve balance recovery responses with minimum predictive gait alterations
Source: PLoS One. 2018 Sep 18;13(9):e0202913. doi: 10.1371/journal.pone.0202913 (PMC6143193; doi:10.1371/journal.pone.0202913)
Supplement: S2 Appendix — (PDF) [file pone.0202913.s002.pdf]

S2 Appendix. Summary of the study hypotheses and corresponding statistical analyses

Study hypotheses	Statistical analysis
Primary:	
[Pr1] Exposure to perturbations with increasing unpredictability can improve balance recovery responses	[Pr1] Improvements in balance recovery responses were assessed by examining whether changes existed in recovery-step kinematics at the completion of the condition using random hazard locations (S1 vs S12 and T1 vs T12).
[Pr2] in the absence of predictive gait alterations.	[Pr2] To confirm the absence of predictive gait alterations at the end of condition 3, kinematics between the first and last non-perturbed trials (N1 vs N6), and previous-step kinematics between the first and last trials for slips (S1 vs S12) and trips (T1 vs T12) were compared.
Secondary:	
[Se1] Predictive gait alterations (e.g. shift of CoM) acquired through exposure to perturbations at a fixed condition	[Se1] Predictive gait alteration was first assessed by examining whether changes existed in gait parameters during regulated non-perturbed trials (N1 vs N2), and secondly, change in previous-step kinematics during slip/trip trials at the fixed hazard locations (S1 vs S4 and T1 vs T4).
[Se2] would not transfer to conditions where both the type and location of perturbation were randomly presented and highly unpredictable.	[Se2] If significant changes (i.e. predictive gait alterations) were observed, transfer between limbs and locations was assessed by investigating whether the changes persisted when a condition was changed without notice (inter-limb transfer: N1 vs N3, S1 vs S5 and T1 vs T5, inter-location transfer: N1 vs N5, S1 vs S9 and T1 vs T9).

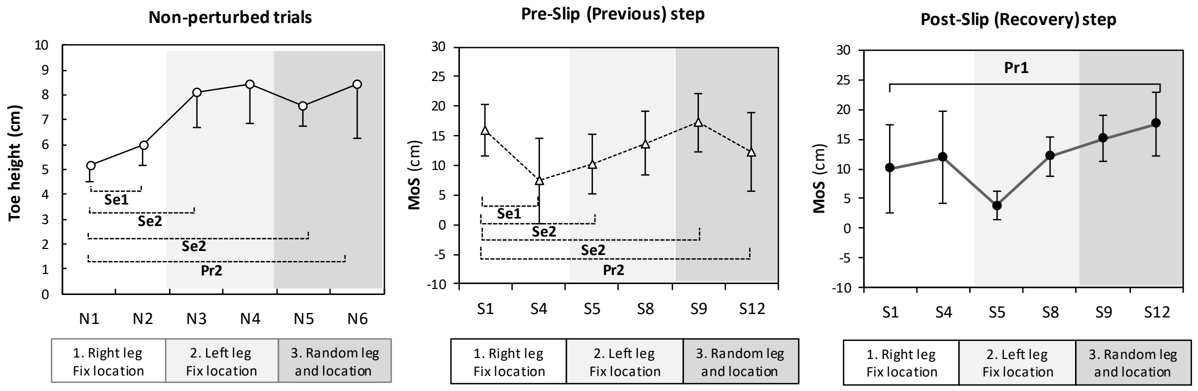

The figures were extracted and modified to indicate the comparisons corresponding to the primary (Pr) and secondary (Se) hypotheses.
